# Experimental Testing of Bionic Peripheral Nerve and Muscle Interfaces: Animal Model Considerations

**DOI:** 10.3389/fnins.2019.01442

**Published:** 2020-01-30

**Authors:** Martin Aman, Konstantin D. Bergmeister, Christopher Festin, Matthias E. Sporer, Michael Friedrich Russold, Clemens Gstoettner, Bruno K. Podesser, Alexander Gail, Dario Farina, Paul Cederna, Oskar C. Aszmann

**Affiliations:** ^1^Clinical Laboratory for Bionic Extremity Reconstruction, Department of Surgery, Medical University of Vienna, Vienna, Austria; ^2^Division of Biomedical Research, Medical University of Vienna, Vienna, Austria; ^3^Otto Bock Healthcare Products GmbH, Vienna, Austria; ^4^Cognitive Neuroscience Lab, German Primate Center, Göttingen, Germany; ^5^Department of Bioengineering, Imperial College, London, United Kingdom; ^6^Section of Plastic and Reconstructive Surgery, Department of Surgery, University of Michigan, Ann Arbor, MI, United States; ^7^Division of Plastic and Reconstructive Surgery, Medical University of Vienna, Vienna, Austria

**Keywords:** bionic prostheses, interface, animal model, testing, electrode, animal

## Abstract

**Introduction:** Man-machine interfacing remains the main challenge for accurate and reliable control of bionic prostheses. Implantable electrodes in nerves and muscles may overcome some of the limitations by significantly increasing the interface's reliability and bandwidth. Before human application, experimental preclinical testing is essential to assess chronic *in-vivo* biocompatibility and functionality. Here, we analyze available animal models, their costs and ethical challenges in special regards to simulating a potentially life-long application in a short period of time and in non-biped animals.

**Methods:** We performed a literature analysis following the PRISMA guidelines including all animal models used to record neural or muscular activity via implantable electrodes, evaluating animal models, group size, duration, origin of publication as well as type of interface. Furthermore, behavioral, ethical, and economic considerations of these models were analyzed. Additionally, we discuss experience and surgical approaches with rat, sheep, and primate models and an approach for international standardized testing.

**Results:** Overall, 343 studies matched the search terms, dominantly originating from the US (55%) and Europe (34%), using mainly small animal models (rat: 40%). Electrode placement was dominantly neural (77%) compared to muscular (23%). Large animal models had a mean duration of 135 ± 87.2 days, with a mean of 5.3 ± 3.4 animals per trial. Small animal models had a mean duration of 85 ± 11.2 days, with a mean of 12.4 ± 1.7 animals.

**Discussion:** Only 37% animal models were by definition chronic tests (>3 months) and thus potentially provide information on long-term performance. Costs for large animals were up to 45 times higher than small animals. However, costs are relatively small compared to complication costs in human long-term applications. Overall, we believe a combination of small animals for preliminary primary electrode testing and large animals to investigate long-term biocompatibility, impedance, and tissue regeneration parameters provides sufficient data to ensure long-term human applications.

## Introduction

Complex prosthetic reconstruction has become a standard for providing patients with useful extremity replacement. However, intuitive control of multiple degree of freedom myoelectric prostheses has proven to be a difficult goal (Aszmann et al., 2015; Hruby et al., 2017). Therefore, a main goal of current research has been to improve the man-machine interface, which remains the main challenge for intuitive control thus causing high abandonment rates (Biddiss and Chau, 2007). In this undertaking, the realistic and chronic simulation of a long-term application is a key aspect to any successful development, which requires experimental testing in animal models.

Since its introduction in the 1940s, the standard approach to interfacing is the use of few muscle-control signals recorded by surface EMG electrodes (Bergmeister et al., 2017). With this classic approach, two signals are used to control multiple degrees of freedom, often resulting in non-intuitive control (Farina and Aszmann, 2014). Prosthesis control is achieved by mapping signals derived from the patients' muscles or nerves into prosthetic motions. Sensory feedback is obtained by converting sensor information from the prosthesis into signals detectable by the human body. For both control and sensory feedback, standard surface electrodes suffer significant limitations, which may potentially be solved by implantable interfaces (Ortiz-Catalan et al., 2012; Farina and Aszmann, 2014). With fairly new surgical procedures, such as targeted muscle reinnervation (TMR) and regenerative peripheral nerve interfaces (RPNI), more muscle signals can be created and, as recent findings show, more cognitive input can be extracted from muscles after targeted reinnervation (Urbanchek et al., 2016; Frost et al., 2018; Vu et al., 2018; Bergmeister et al., 2019). Accordingly, new interfaces are essential to deal with this surplus of potential motor control signals (Bergmeister et al., 2017). Modern multichannel electrodes show promising results for high resolution recording of multiple muscle signals after TMR, but surface-electrode related issues are still present (Kapelner et al., 2016). Therefore, modern interfacing concepts focus on developing a stable broadband interface for rapid communication between patient and prosthesis. Hereby, several approaches are used, including implantable electrodes connected to muscle or nerve tissue to optimize signal quality for control and feedback. This approach becomes particularly interesting with fully implantable devices using wireless data transmission, through the skin, since this avoids percutaneous cables with their associated infection risks and aesthetic concerns (Weir et al., 2009; Pasquina et al., 2015; Bergmeister et al., 2016b). Percutaneous leads have been used for diaphragm pacing for decades as a life-saving procedure, thus justifying the above-mentioned risks. Controversially, for prosthetic control the risk-benefit-ratio may not be as favorable, since the available 1–2 channels are insufficient (Onders et al., 2018).

Implantation of medical devices requires extensive preclinical testing to analyze long-term biocompatibility as well as functionality of the implant (del Valle and Navarro, 2013; Pasquina et al., 2015; Bergmeister et al., 2016b). Every device needs to be tested experimentally for regulatory accreditation as a medical implant. Thus, animal models are necessary to test safety and efficacy prior to human implantation. Finding the right animal model for testing interfacing devices is challenging, as some devices require muscle signals from natural moving animals and comparable mechanical stress to predict human application.

When choosing an animal model, behavioral characteristics should be considered, as frequent handling of the animals during testing is often required. Costs and availability as well as ethical considerations and legality play also a major role when choosing the right model. To quote Bernard E. Rollin (Rollin, 1990): “*The most brilliant design, the most elegant procedure, the purest reagents, along with investigator talent, public money, and animal life are all wasted if the choice of animal is incorrect.”*

The aim of this study was to evaluate animal models for muscle/nerve implants considering behavioral, economical, and ethical aspects in addition to the scientific validity of the model. In addition, we present an overview of models used in the current literature. Based on our analyses, we describe an algorithm for experimental testing of fully implantable devices under natural moving conditions and their advantages and disadvantages.

## Methods

### Systematic Literature Analyses

**Figure 1:**
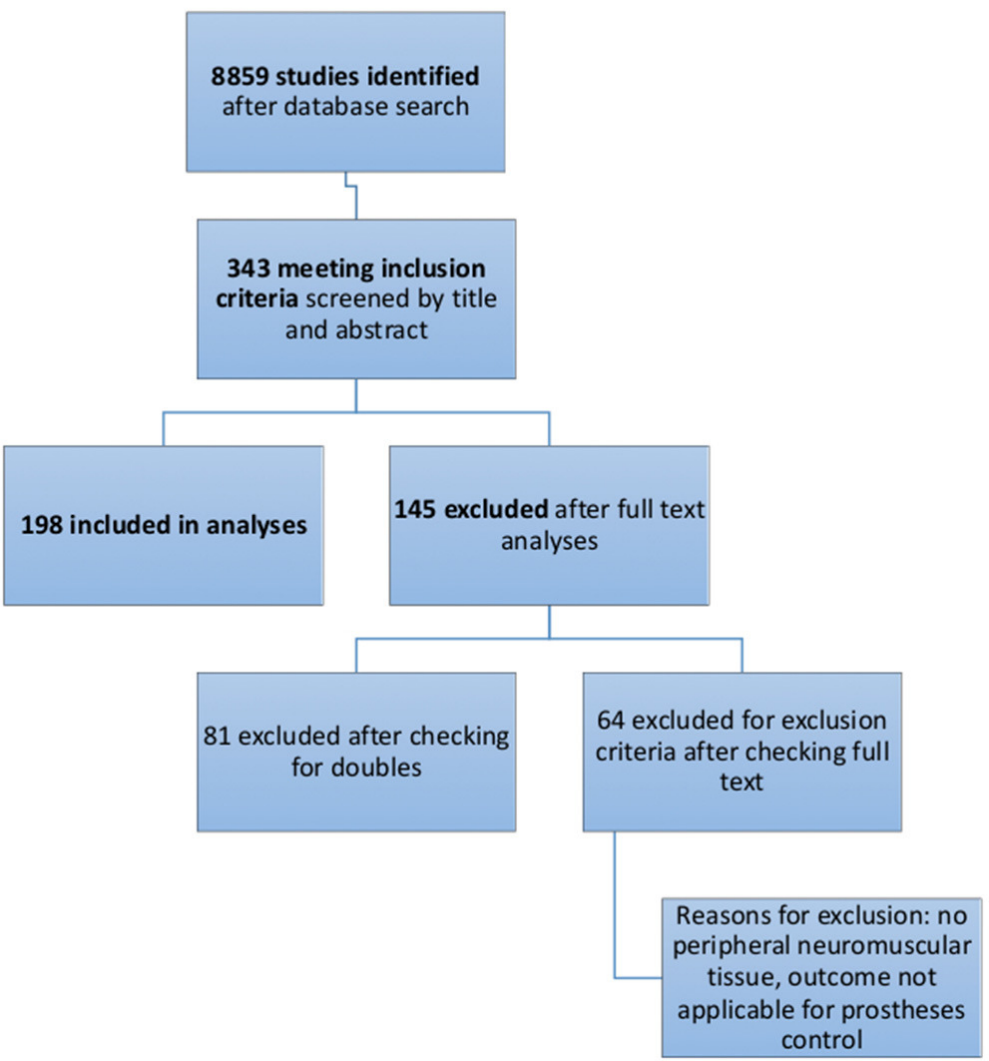
Flow diagram of the selection process of systematic literature analyses, as recommended by PRISMA Guidelines.

The authors designed a systematic search strategy for PubMed and Google Scholar according to the PRISMA guidelines (Liberati et al., 2009) (Figure 1). Potentially relevant studies were screened for inclusion criteria, which included all studies describing animal models for testing bionic interfaces or electrodes connected to peripheral nerve or muscle. The date of the last entry for each database was December 31st, 2017. The results of this systematic search were screened for possible inclusion against a predetermined PICOS checklist of inclusion criteria (Table 1). All publications using animal models in English and German were included. Two levels of screening were used on the citations. First, titles and abstracts were screened to identify all potentially eligible studies. Studies meeting the inclusion criteria were obtained in full text and assessed thoroughly for eligibility. Additionally, the reference lists of the included literature were used to identify further relevant publications. Two reviewers independently applied the criteria for inclusion in reviewing the retrieved articles (Supplementary Material). If any differences were perceived toward inclusion, they were resolved by discussion among the authors.

**Table 1:** This table shows the PICOS (Patient, Intervention, Comparison, Outcome, Study design) inclusion criteria, used for systematic literature search.

**PICOS**	**Inclusion criteria**
Population	Animal Models
Intervention	Implantation of interfacing device or electrode interacting with peripheral neuromuscular tissue Peripheral Nerve Muscle tissue
Comparison	Animals used Duration Group size Advantages of various models
Outcome	Type of animal Group size Duration Origin of publication Costs and ethics (if mentioned)
Study design	Experimental animal study English or German language

### Economic and Ethical Evaluation of Standard Models

All models were analyzed for costs on the basis of the in-house prices of the Center of Biomedical Research at the Medical University of Vienna. For international comparison, relative relations are given to compare among different models. Ethical standards according the FELASA principles were obtained (Guillen, 2012).

The most common nerve model, the rat sciatic nerve was evaluated for feasibility at our facility, comparing operation time to accessing the sciatic nerve between surgeons and unexperienced academic staff. This was performed to proof the model as performable for non-surgeons.

### Animal Trials

All animal models from the authors' institution were conducted with permission of the ethics committee of the Medical University of Vienna and the Austrian Ministry for Research and Science (BMWF: reference number: BMWF-66.009/0309-WF/II/3b/2010, BMWF-66.009/0340-WF/II/3b/2016, BMWF-66.009/0024-WF/II/3b/2018).

## Results

### Literature Analyses

**Figure 2:**
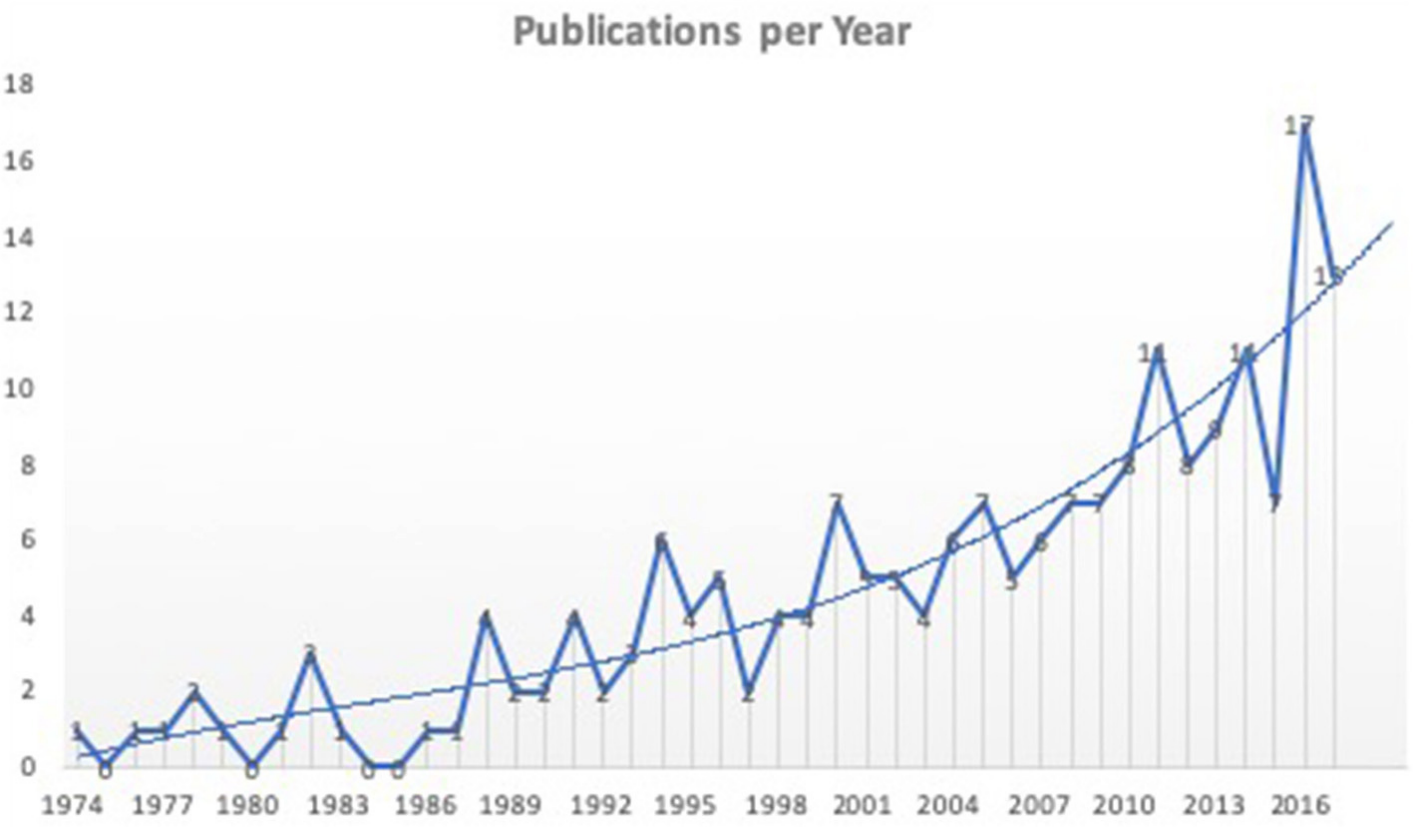
Literature analyses showed an upward trend for publications of new interfacing devices. Shown are all 198 studies included in the final analyses according to the year of publication. Not all studies represent new interfacing devices, but rather studies using animal models to test electrodes or stimulation parameters for interfacing bionic prostheses.

A total of 8859 studies were identified with the search terms listed in Table 2. Thereof 343 were included after title and abstract evaluation. Of these, 81 publications were excluded after checking for double results and another 64 publications were excluded after screening the full text. Exclusion criteria are listed in Figure 2. A total of 198 were included in the final analyses.

**Table 2:** Search terms for the systematic literature search.

**Search terms**
Animal AND implantable AND electrode
Animal AND peripheral AND electrode
Animal AND EMG AND prostheses
Implantable EM
Animal model AND prostheses AND control
Animal model AND extremity AND reconstruction
Animal model AND EMG test
Animal model AND electrode testing

Analyses showed a significant trend toward the development and testing of interfacing devices over the last years.

Most published studies originated from Northern America (55%) and Europe (34%). Studies from Asia and Australia accounted for 10 or 1%, respectively. South America and Africa were not represented in the identified studies.

**Figure 3:**
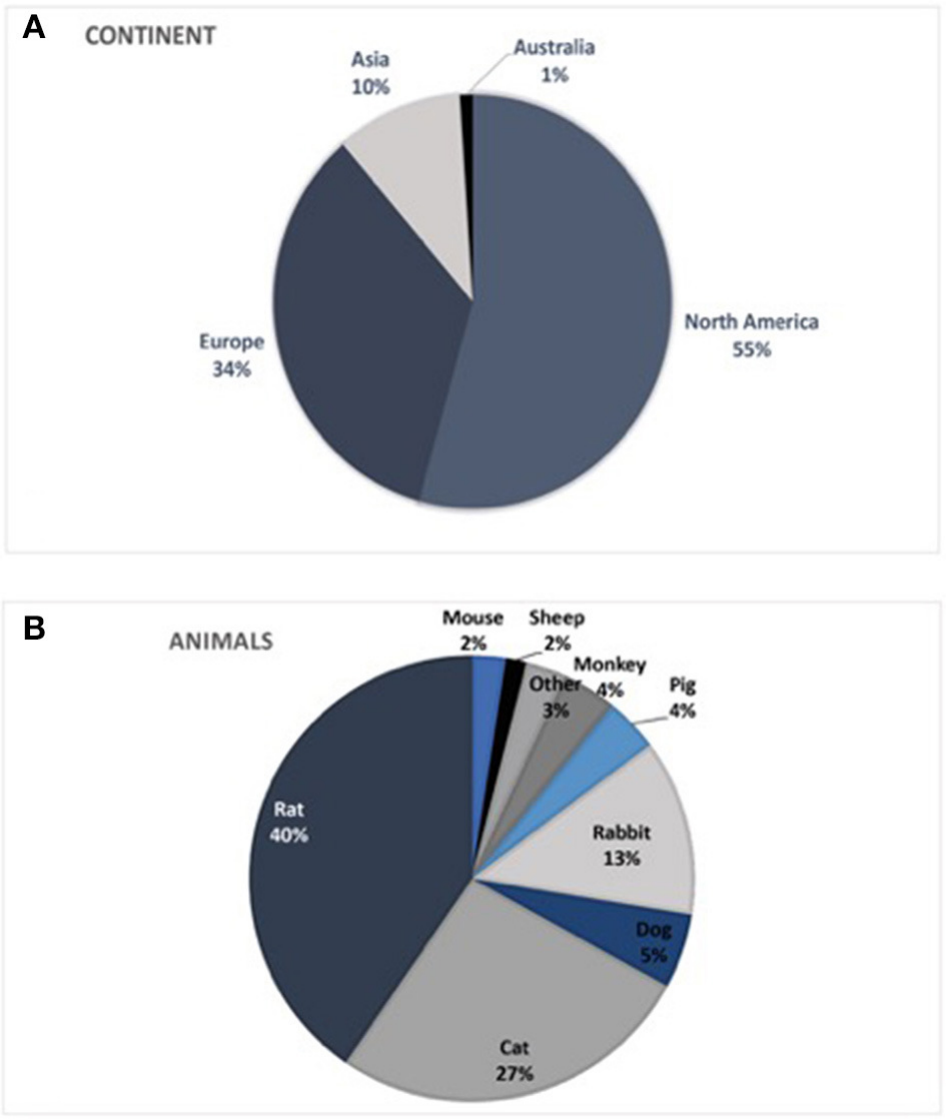
**(A)** Origin of publications by continent. South America and Africa were not represented in publications identified in the literature search. **(B)** Animal models used worldwide in testing bionic interfaces, indicated by the literature analyses.

**Figure 4:**
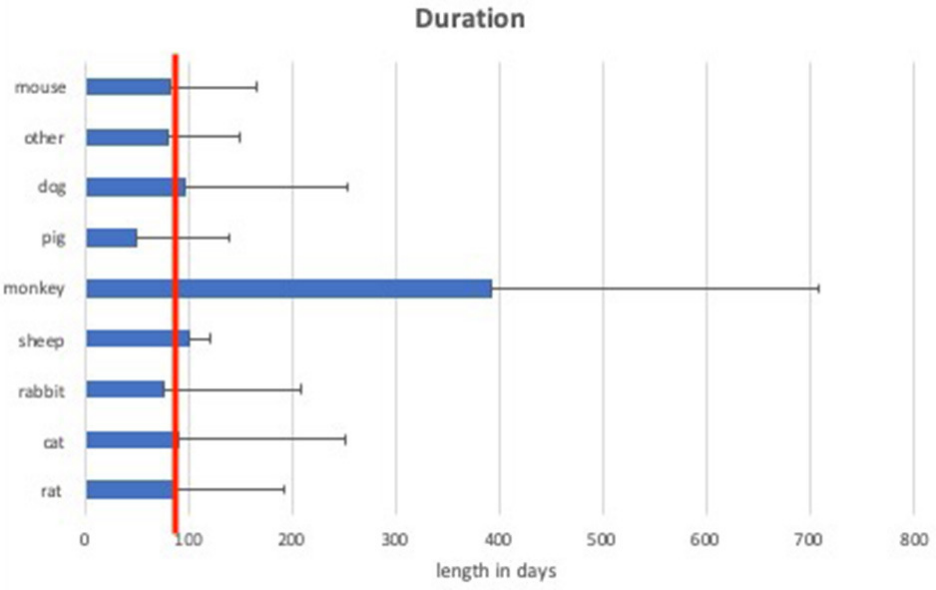
Mean duration of experiments of different animal models. The red line indicates 90 days of duration, which is considered as long-term (chronic).

Analyses of the animals used in the literature showed predominant use of the rat model (40%). Rat trials had a mean duration of 87 ± 105 days (range: 1–390 d) with a mean sample size of 12.5 ± 10.3 animals (range 1–51) per study. Cats were used in 27% of the studies with a duration of 92 ± 158 days (range: 1–900 d) and a mean of 6.9 ± 4.8 animals (range 1–26) per study. Rabbit trials accounted for up to 13% with 11.9 ± 10.2 animals (range 1–40) and 77 ± 132 days (range 1–480 d). Dog models were used in 5% of the publications with 3.3 ± 2.8 animals (range 1–10), analyzed over 97 ± 157 days (range 1–450 d). Pigs [5.3 ± 3.3 animals (range 1–11), 50 ± 88 d (range 1–270 d)] and monkeys [1.8 ± 0.9 animals (range 1–4), 394 ± 314 days (range 1–930 d)] were both used in 4% of the studies. Sheep [2.5 ± 1.5 animals (range 1–4), 102 ± 18 days (range 84–120 d)] and mouse models [12.3 ± 6.8 animals (range 7–22), 84 ± 83 days (range 16–200 d)] only accounted for 2% each. The remaining 3% were made up from unique experiments including raccoons, guinea pigs, frogs, crayfish, and zebrafish (Figures 3, 4).

Large animal models (rabbit, cat, dog, monkey, pig, and sheep) had a mean duration of 135 ± 87.2 days compared to small animal models (mouse, rat) with a mean duration of 85 ± 11.2 days.

Considering the length of the trials, experiments with a duration over 3 months (90 days) are defined as chronic (long-term) experiments (Anderson et al., 2008; Parasuraman, 2011). According to this classification, 37.5% of rat, 33.3% of mouse, 38% of cat, 28% of rabbit, 100% of sheep, 75% of monkey, 25% of pig, and 20% of dog experiments were chronic experiments. This accounts for a total of 37.2% of small animals and 36.9% of large animal models being chronic experiments.

Muscular interfaces were tested in only 23% of the studies, thus the vast majority of the selected publications related to neural interfacing (77%). Here, 96% of cats were used for neural interfaces as well as 59.6% of dogs, 89% of pigs, 88% of rabbits, 79.2% of rats, 0% of sheep, and 33.3% of primates. For the rat model, which was the most common animal model used for neural interfacing, 88.5% of the studies focused on neural interfaces using the sciatic nerve. Of the remaining studies on the rat, 11% used the vagal and glossopharyngeal for sensory recordings or stimulation.

### Sciatic Nerve Preparation

To evaluate feasibility of the most commonly used peripheral nerve model in the rat—the sciatic nerve model—we compared the procedure between surgeons and academic staff with low surgical experience at the Center of Biomedical Research of the Medical University of Vienna. The average time from skin incision to accessing and preparing the sciatic nerve, was 2 min ± 35 s for surgeons compared to 3 min ± 40 s for academic staff.

### Cost Analyses

**Figure 5:**
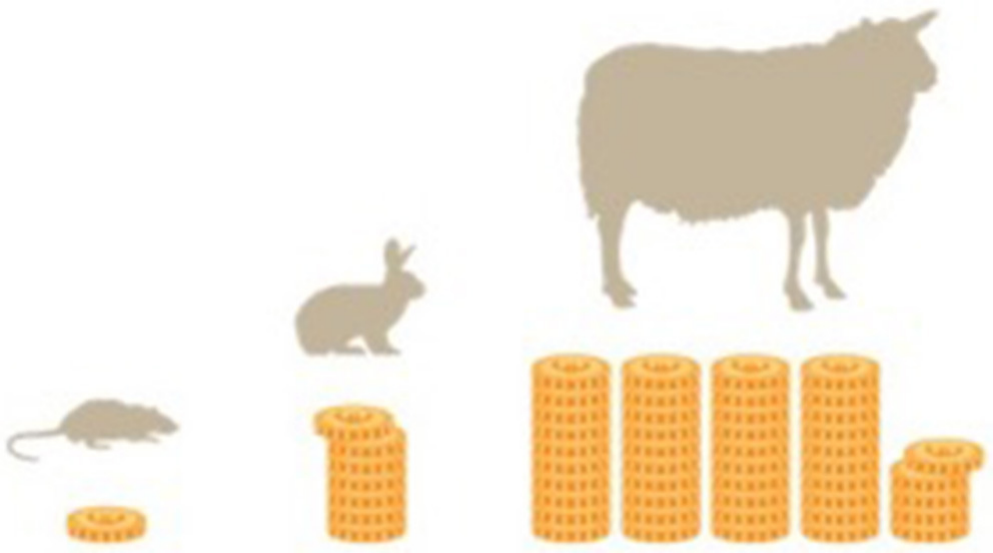
Relative proportions of the costs of different models are given. The rabbit is by a factor 7.2 more expensive than a rat model. Sheep models are up to a factor 45.5 more expensive than rat models.

Costs analyses are based on in house costs at the Center for Biomedical Research at the Medical University of Vienna, which significantly varied among different models. Acquisition costs and overall costs of operation (including anesthesia, analgesia, consumption items, instrument sterilization, and required staff costs) for a single animal where ~110€ (124$) for a rat, 800€ (900$) for a rabbit, and up to 5,000€ (5,640$) for a large model like pig or sheep. These costs do not include housing after the operation which varies from 90 Cent (1$) per day for a rat, over 3.75 € (4.23$) per day for a rabbit, and up to 6.5 € (7.33$) for a sheep. Large animal models are therefore more expensive than the rat model by a factor of 7.2 (rabbit) and 45.4 (sheep). Costs were analyzed on the basis of costs of representative trials over the last years. Other institutional charges, such as acquisition or housing costs may vary in different countries, but relative relations should remain comparable (Figure 5).

Cost analyses of a primate model at the DPZ (German Primate Center) show acquisition costs only of up to 8,000€ (9,080$). As operation requires a team of several people involved, costs for a single animal as calculated above increase tremendously. Housing costs account up to about 25€ (28.37$) per day.

## Discussion

Although modern bionic prostheses are technically very advanced and capable of complex movements, the interface between man and machine is still a limiting factor for sophisticated control. Therefore, research efforts are directed into the development of implantable interfaces, which is evident in the upward trend of recent publications (Figure 3). The analysis of new devices requires extensive animal testing to ensure long-term safety for human application. Hereby, choosing the right animal model is essential to ensure sufficient and reliable data as certain analyses predetermine the required model. Grasping tests require animals with fingers, whereas walking analyses require non-cloven hoof animals. Only few models (mice, rats) can be genetically modified if necessary. Life span varies among different animals as well as regeneration and handling aspects, impeding the choice of animal. This study therefore evaluates current models in the literature and describes an algorithm for international standardized testing.

The most commonly used model for *in vivo* testing is the rat model. A rat is large enough to perform various surgeries, with the benefit of cheap asset costs and housing. Rats show excellent regeneration as well as favorable immunological properties which is beneficial for implant testing. Despite minor immunologic differences between rodents and humans, biocompatibility is often assessed in rats to give a prediction for human use (Anderson and McNally, 2011). However, it is generally suggested to provide additional studies in large animals (Mestas and Hughes, 2004), as for example, rats have a faster axonal regeneration than humans (2–3 vs. 1–2 mm/day) as well as robust axonal sprouting, hampering direct clinical translation of this model. From an ethical point of view, as in all animal trials, animal welfare during the trial must be ensured. Furthermore local regulations must be obtained as for example the 3R principle or regulations obtained by the FELASA (Rehbinder et al., 1996; Guillen, 2012). As mentioned before, rodents are a frequently used animal models without severe ethical issues in most countries.

Rat handling is comparably easy. It requires basic training and can be accomplished even in small facilities. The rat offers many possibilities for both muscular and neural interface testing due to easy accessible nerves, such as the sciatic nerve, which is considered the gold standard (Vasudevan et al., 2017). It is the largest nerve in the rat, easily accessible with a single incision between the biceps femoris and the gluteus superficialis muscle. This was also shown in our comparison between surgeons and academic staff with low microsurgical skills, which clearly showed that the rat sciatic nerve model is easy to master even for non-surgical staff. Post-operative complications in the rat model are rare and include wound dehiscence or self-mutilation in case of nerve damage or transection of the nerve. Other surgical complications, such as hematoma or seroma formation are common complications and can affect all animal models undergoing surgery. Despite the lower extremity being the gold standard, rat trials are not only limited to the sciatic nerve. As the vast majority of bionic prostheses target upper extremity amputations, the forelimb of the rat has also been accessed to simulate a more realistic final use. Here, the brachial plexus offers a variety of nerves. For a detailed description and surgical approach we refer to our previous work (Bergmeister et al., 2016a). We have also recently shown the feasibility of using multichannel EMG electrodes in a rat TMR model. This model has provided reliable data on acute electrode implantation and recording, and on the combination of TMR and implantable electrodes, thus simulating the actual clinical application (Muceli et al., 2018).

Besides the common use of the rat model for neural implants, muscle electrodes can also be tested with this model. Our literature analyses showed that 20.8% of the studies used the rat model to evaluate muscular interfaces.

Despite many advantages of the rat model, for human-sized implants this model is promptly limited. Implantable interfaces, connected to muscle or nerve tissue, are subjected to high mechanical stress due to movements and muscle contractions. To simulate this mechanical stress, large animal models are advisable, which furthermore eases fitting of the implant due to comparable anatomy to humans.

Many of these requirements can be achieved in a rabbit model, which is ~7 times more expensive than a rat model, but still significantly cheaper than sheep or pigs. Also, the rabbit has the advantage of easy handling compared to larger animals. The sciatic nerve in the rabbit is, with some experience and knowledge of anatomical landmarks, as accessible as in the rat, but significantly larger. A big disadvantage of the rabbit model, however, is the high susceptibility to infections, which is especially relevant for long-term studies with implants. Sterility during the procedures is vital and strict post-operative care as well as antibiotic prophylaxis obligatory. Self-mutilation is frequent and implies high dropout rates. Appropriate suturing and animal cones as well as environmental enrichment can decrease stress for the animals and thereby the dropout rate (Wheeler et al., 2015). Blood counts, required for biocompatibility studies, can easily be sampled due to the prominent vein in the rabbit's ear. Further care must be taken when handling the animal as rabbits are frail for injuries of the spinal cord. Again, most countries allow testing devices in rabbits. A lot of attention in the media due to testing of the cosmetic industry in rabbits was present resulting in some disagreeability in this model. Still it is a valid and commonly used model worldwide.

A significantly larger and therefore more robust animal is the sheep, which has the advantage of allowing implanting devices of real size for human application (Sartoretto et al., 2016). Sheep models have been gaining popularity worldwide and the FELASA, the Federation of European Laboratory Animal Science Associations, even suggests replacing dog trials with sheep (Rehbinder et al., 2000). Care must be taken while anesthesia, as sheep are ruminants with a certain risk of aspiration. Due to their body size and weight, sheep are easier to handle than pigs, especially in long-term experiments. In case of EMG signal assessment, when normal gait behavior is required, difficulties may arise for data acquisition, as sheep are not used to perform certain tasks or exercises. In one of our previous studies, we have performed an experiment in sheep with an implantable telemetry system (Bergmeister et al., 2016b). For that purpose, we designed a custom saddle for sheep to place the telemetry unit over the implant and to be able to acquire signals when the animals were moving freely. After the implantation of the system, sheep had a post-operative phase at the facility where they were checked daily for pain, infection, wound healing and possible complications. After recovery was ensured, the sheep were transferred to an outsourced facility with a pasture, where all animals could move freely. With a specialized animal trainer, various gait behaviors were trained to get repeated measurements of certain muscle activity. Even though an inductive system was tested, signals could easily be acquired with the custom-made saddle carrying the external control device (Bergmeister et al., 2016b).

Similar to sheep, pigs offer comparable anatomy—especially regarding size- to the human body but with thicker skin and consequently possible influences on data transmission for telemetry studies. Weight gain has to be considered, especially in long-term experiments, as this can induce higher mechanical stress on implants compared to humans. Also, daily handling of the animals is hampered by their weight, particularly if frequent measurements are needed in awake animals. But even anesthetized animals require more trained staff for handling than other animals.

Easier to handle are cats and dogs, which have been widely used especially in Northern America. Behavioral training to perform certain tasks and reproducible gait behavior are advantages of these models, which can be achieved with less effort than with other models. Contrary to their popularity in Northern America, some European countries have banned these models, or restricted surgical interventions in these models. Therefore, international reproducibility and comparability is limited.

Walter et al. (1996) described the raccoon as model for device evaluation. Raccoons have superior grasping ability, compared to all other non-primate animal models. However, availability, possible difficulties in housing and handling as well as legal considerations are reasons for the very limited use of this model.

Very close to human nature is the *in vivo* evaluation in a non-human primate (NHP) model. NHPs are the only experimental animals capable of purposive movements with the same biomechanics as a human, except for the opposable thumb (Baker et al., 2008). Cognitive function as well as cerebral representation of involved structures are comparable and higher primates are the only animals that share with humans the anatomy of direct neuronal projections from cerebro-cortical to spinal motor neurons for control of dexterous movements of distal extremities (Lemon, 2008). Contrary to other animal models, NHPs, especially rhesus monkeys but the certain extend also marmoset monkeys, can be well-trained to perform complex motor tasks repeatedly and reliably upon instruction. NHPs are able to perform a large number of comparable movements, making the acquired data particularly valuable for later statistical analysis. Hereby, more than 100 repetitions can frequently be obtained in a single session. Monkeys can be trained to perform very precise movements with different instructed and controlled movement kinematics and kinetics. In rhesus monkeys, the upper arm muscles are of similar size as the forearm muscles of humans, making them ideally suited for the assessment of bionic control interfaces, especially for reach and grasp movements (Baker et al., 2008). Smaller NHP models, like marmoset monkeys, lack comparable muscle size, limiting, for example, the comparability in terms of mechanical stress on the implants.

The use of complex tasks involving dexterous movements, for which the primate model is ideally suited, comes with costs, though. Training for a complex experiment needs to be done extensively and new tasks require re-training. For complex tasks, animals need to be continuously trained to maintain their level of performance. Training is mostly needed for making use of the high number of precisely repeated movements and the experimentally well-controlled timing, though, which in other animal models is not even an option. This training effort can be reduced with increasing naturalism of the behavior to be studied, e.g., walking in a freely moving animals (Berger and Gail, 2018) provided wireless transmission over long enough distances or data logging for EMG signals is available. Particularly in NHPs, since they have large degree of freedom for arm movements and precise grasp behavior, external devices need to be well-protected and outside the reach of the animals. This can create a challenge for external data transmission components that are frequently required. Due to the very high standards for keeping monkeys they are expensive compared to other experimental animals and a cost-benefit analysis is advised before engaging in work with NHPs. For economic and ethical reasons, the NHP model should only be used when the implant development is far enough advanced so that the particular benefits of this model, like complex movement behavior and similarity to human biomechanics, are scientifically mandatory. Therefore, mainly final stage devices are tested in NHPs before human application, such as the IMES or MyoPlant systems (Baker et al., 2010; Morel et al., 2016). Finally, the use of NHPs is restricted in several countries.

Lastly, testing of feedback devices or electrodes for sensory feedback remains difficult in animal models, although it has already been demonstrated in human studies (Flesher et al., 2016). Biocompatibility as well as electromechanical properties of the device can be tested in a model of adequate size. Device functionality can only be tested indirectly via loop control or pre-determined signs, such as muscle twitches, as no animal model is capable of fully providing feedback sensations. There are studies for successful report of stimulation of somatosensory cortex in NHPs, which might be a way to image feedback devices with fMRI. This option however, is cost-intensive and availability is highly limited (Wu et al., 2017). Findings from Flesher et al. demonstrated recordability of evoked signals from the cortex, which also could be translated into animal trials as a possibility to obtain feedback information from peripheral stimulation.

Our findings that only about 37% of the trials exceeded more than 3 months duration seems alarming for long-term safety of patients (Figure 4). Although sometimes accelerated failure models are used to simulate long term application (e.g., increasing stimulation frequency to simulate years of stimulation within weeks), care should be taken to get safe products that resist mechanical stress as well as biological environments with humidity and foreign body reaction (Anderson et al., 2008). Different surveys worldwide show an average age in the range 25–36 years for patients at the time of upper extremity amputation, revealing not only the demand for bionic reconstruction, but furthermore the demand for devices that last for decades (Jang et al., 2011; Craig et al., 2017). There is a huge variety of materials used for medical implants. Some of them have been used for decades in human implants (e.g., cardiac pacemakers). Therefore, when testing a new device with parts of commonly used and previously tested materials, a focus should be on mechanical stability of the implant (Boutrand, 2019). New trends in material science use immunohistochemical analyses for assessing biocompatibility and show a trend toward flexible and individual implants (Song et al., 2019).

The costs of larger animals are significantly greater compared to smaller animals. This fact should not result in fewer animals per trial and shorter durations of the trials. Ultimately, the costs of preclinical trials are minor, compared to the potential costs of complications, device failure, and patient's burden (Fuller et al., 2009).

### Testing Approach

*In vivo* evaluation of bionic interfacing devices requires biocompatibility studies, mechanical stability as well as functionality in terms of chronic data acquisition. We recommend starting in a rat model to assess the system or individual parts. Thereby, devices can be refined if necessary, with a considerably smaller financial burden or time and labor. Implantation in the rat is followed by a post-operative phase of 3–5 days with analgesia and can be combined with antibiotics to prevent infections. Different time points can be assessed which are mainly 4 and 12 weeks and sometimes 6 months for long-term applications (Anderson et al., 2008). Hereby important factors such as biocompatibility, mechanical safety of the electrodes, and impedance can be reliably assessed. We also recommend a stage wise approach by evaluating the device in a 4-week group and only starting with the next step (e.g., 12-weeks group) if no adverse events occur. This approach saves time and money and minimizes animal burden if the devices do not function as expected.

Implantation of a full-sized system requires larger animals. As dogs are not available everywhere, international comparability among new interfaces would benefit from standardized models. Depending on the size of the implant, either a rabbit or sheep model should be used. A rabbit model is cheaper and easier during daily handling, in contrast to the sheep, which instead has advantages of fitting large implants. Anatomical size of involved nerves and muscles, as well as biomechanical stress to the implant is more realistic in the sheep model, which therefore is generally recommended. A large animal trial for a device for clinical application must at least have a duration of 3 months to be considered long-term (Anderson et al., 2008; Anderson and McNally, 2011; Anderson, 2015).

Final testing could be conducted in an NHP model if the complexity of the interface requires this model (brain interfaces or peripheral interfaces with a large number of degrees of freedom for high-DOF hand prostheses, for example). However, for standard electrodes, a long-term application in a standard large animal (sheep) provides enough data on biocompatibility and mechanical stability (Bergmeister et al., 2016b; Sartoretto et al., 2016) for human translation.

## Conclusion

Testing of new devices is important to improve the components of future bionic interfaces. To ensure patient safety, and to prevent complications in long-term use of novel devices, extensive preclinical testing is crucial. For this purpose, various animal models are available and can be used over different time points. As it is of utmost importance to find the right model for preclinical evaluation, we provide an overview of existing models, and describe the common approach for testing new implants. An iterative approach should be used from short-term rat models, to assess compatibility, safety and functionality of the implant, up to long-term large animal models, such as sheep, to evaluate data acquisition as well as long-term safety. International comparability is granted by a sheep model but not for cat and dog models, as these models are not available in every country. An NHP model can be chosen for complex trials, however limited availability due to ethical concerns as well as costs must be taken into account.

The ultimate goal of testing new interfacing devices is the implantation in humans for sophisticated prosthetic control and feedback, which should last for a lifetime and therefore be tested extensively in the correct animal model for biological and mechanical safety.

## Data Availability Statement

The datasets used and/or analyzed during the current study are available from the corresponding author on reasonable request.

## Ethics Statement

All animal models from the authors' institution were conducted with permission of the ethics committee of the Medical University of Vienna and the Austrian Ministry for Research and Science (BMWF: reference number: BMWF-66.009/0309-WF/II/3b/2010, BMWF-66.009/0340-WF/II/3b/2016, BMWF-66.009/0024-WF/II/3b/2018).

## Author Contributions

MA, KB, and OA designed the concept. All authors analyzed data, contributed their specific expertise, wrote the manuscript, and revised it critically.

### Conflict of Interest

MR was employed by the company Otto Bock. The remaining authors declare that the research was conducted in the absence of any commercial or financial relationships that could be construed as a potential conflict of interest.

## Abbreviations

FELASA: Federation of European Laboratory Animal Science Associations
NHP: Non-human primate.
