# Supplementary material for: Experimental Testing of Bionic Peripheral Nerve and Muscle Interfaces: Animal Model Considerations
Source: Front Neurosci. 2020 Jan 30;13:1442. doi: 10.3389/fnins.2019.01442 (PMC7025572; doi:10.3389/fnins.2019.01442)
Supplement: Supplementary file 1 [file Data_Sheet_1.PDF]

A fully implanted intramuscular bipolar myoelectric signal recording electrode. Neuromodulation. 2014 Dec;17(8):794-9; discussion 799. doi: 10.1111/ner.12165. Epub 2014 Mar 10. Memberg WD1, Stage TG, Kirsch RF.

A multielectrode array for intrafascicular recording and stimulation in sciatic nerve of cats. Brain Res Bull. 2000 Mar 1;51(4):293-306. Branner A1, Normann RA.

A nerve cuff technique for selective excitation of peripheral nerve trunk regions. IEEE Trans Biomed Eng. 1990 Jul;37(7):706-15. Sweeney JD1, Ksienski DA, Mortimer JT.

A slowly penetrating interfascicular nerve electrode for selective activation of peripheral nerves. IEEE Trans Rehabil Eng. 1997 Mar;5(1):51-61. Tyler DJ1, Durand DM.

A spiral nerve cuff electrode for peripheral nerve stimulation. IEEE Trans Biomed Eng. 1988 Nov;35(11):905-16. Naples GG, Mortimer JT, Scheiner A, Sweeney JD.

Action potential classification with dual channel intrafascicular electrodes. IEEE Trans Biomed Eng. 1994 Jul;41(7):609-16. McNaughton TG1, Horch KW.

Analysis of single-unit firing patterns in multi-unit intrafascicular recordings. Med Biol Eng Comput. 1993 May;31(3):257-67. Goodall EV1, Horch KW, McNaughton TG, Lybbert CM.

Chronic histological effects of the flat interface nerve electrode. J Neural Eng. 2006 Jun;3(2):102-13. Epub 2006 Apr 18. Leventhal DK1, Cohen M, Durand DM.

Chronic measurement of the stimulation selectivity of the flat interface nerve electrode. IEEE Trans Biomed Eng. 2004 Sep;51(9):1649-58. Leventhal DK1, Durand DM.

Conduction studies in peripheral cat nerve using implanted electrodes: I. Methods and findings in controls. Muscle Nerve. 1988 Sep;11(9):922-32. Krarup C1, Loeb GE.

Conduction studies in peripheral cat nerve using implanted electrodes: II. The effects of prolonged constriction on regeneration of crushed nerve fibers. Muscle Nerve. 1988 Sep;11(9):933-44. Krarup C1, Loeb GE, Pezeshkpour GH.

Conduction studies in peripheral cat nerve using implanted electrodes: III. The effects of prolonged constriction on the distal nerve segment. Muscle Nerve. 1989 Nov;12(11):915-28. Krarup C1, Loeb GE, Pezeshkpour GH.

Cuff electrodes for chronic stimulation and recording of peripheral nerve activity. J Neurosci Methods. 1996 Jan;64(1):95-103. Loeb GE1, Peck RA.

Development of an implantable myoelectric sensor for advanced prosthesis control. Artif Organs. 2011 Mar;35(3):249-52. doi: 10.1111/j.1525-1594.2011.01219.x. Epub 2011 Mar 3. Merrill DR1, Lockhart J, Troyk PR, Weir RF, Hankin DL.

Early peripheral nerve regeneration after crushing, sectioning, and freeze studied by implanted electrodes in the cat. J Neurosci. 1994 May;14(5 Pt 1):2659-73. Fugleholm K1, Schmalbruch H, Krarup C.

Effects of consecutive slips in nerve signals recorded by implanted cuff electrode. Med Eng Phys. 2008 May;30(4):460-5. Epub 2007 Jun 27. Tong KY1, Rong W, Li L, Cao J.

Electrode array for reversing the recruitment order of peripheral nerve stimulation: experimental studies. Ann Biomed Eng. 2006 Jan;34(1):152-60. Epub 2006 Feb 2. Lertmanorat Z1, Gustafson KJ, Durand DM.

Electroneurographic recordings with polymer cuff electrodes in paralyzed cats. J Neurosci Methods. 1982 Mar;5(3):267-72. Julien C, Rossignol S.

Evaluation of a thin-film peripheral nerve cuff electrode. J Spinal Cord Med. 1995 Jan;18(1):28-32. Walter JS1, McLane J, Cai W, Khan T, Cogan S.

Fabrication and evaluation of conductive elastomer electrodes for neural stimulation. J Biomater Sci Polym Ed. 2007;18(8):1057-73. Keohan F1, Wei XF, Wongsarnpigoon A, Lazaro E, Darga JE, Grill WM.

Functionally selective peripheral nerve stimulation with a flat interface nerve electrode. IEEE Trans Neural Syst Rehabil Eng. 2002 Dec;10(4):294-303. Tyler DJ1, Durand DM.

Generation of unidirectionally propagating action potentials using a monopolar electrode cuff. Ann Biomed Eng. 1986;14(5):437-50. Ungar IJ, Mortimer JT, Sweeney JD.

Implantable electrode for recording nerve signals in awake animals. J Appl Physiol. 1976 Jul;41(1):111-4. Ninomiya I, Yonezawa Y, Wilson MF.

Implantable myoelectric sensors (IMESs) for intramuscular electromyogram recording. IEEE Trans Biomed Eng. 2009 Jan;56(1):159-71. doi: 10.1109/TBME.2008.2005942. Weir RF1, Troyk PR, DeMichele GA, Kerns DA, Schorsch JF, Maas H.

Information contained in sensory nerve recordings made with intrafascicular electrodes. IEEE Trans Biomed Eng. 1991 Sep;38(9):846-50. Goodall EV1, Lefurge TM, Horch KW.

Instrumentation for ENG and EMG recordings in FES systems. IEEE Trans Biomed Eng. 1994 Jul;41(7):703-6. Nikolić ZM1, Popović DB, Stein RB, Kenwell Z.

Longitudinal intrafascicular electrodes in collection and analysis of sensory signals of the peripheral nerve in a feline model. Microsurgery. 2005;25(7):561-5. Li LJ1, Zhang J, Zhang F, Lineaweaver WC, Chen TY, Chen ZW.

Long-term stimulation and recording with a penetrating microelectrode array in cat sciatic nerve. IEEE Trans Biomed Eng. 2004 Jan;51(1):146-57. Branner A1, Stein RB, Fernandez E, Aoyagi Y, Normann RA.

Muscle recruitment with intrafascicular electrodes. IEEE Trans Biomed Eng. 1991 Aug;38(8):769-76. Nannini N1, Horch K.

Muscle-selective block using intrafascicular high-frequency alternating current. Muscle Nerve. 2010 Sep;42(3):339-47. doi: 10.1002/mus.21678. Dowden BR1, Wark HA, Normann RA.

Neural morphological effects of long-term implantation of the self-sizing spiral cuff nerve electrode. Med Biol Eng Comput. 2001 Jan;39(1):90-100. Romero E1, Denef JF, Delbeke J, Robert A, Veraart C.

Neural prostheses: electrophysiological and histological evaluation of central nervous system alterations due to long-term implants of sieve electrodes to peripheral nerves in cats. IEEE Trans Neural Syst Rehabil Eng. 2008 Jun;16(3):223-3

Non-invasive measurement of the input-output properties of peripheral nerve stimulating electrodes. J Neurosci Methods. 1996 Mar;65(1):43-50. Grill WM1, Mortimer JT.

Non-invasive method for selection of electrodes and stimulus parameters for FES applications with intrafascicular arrays. J Neural Eng. 2012 Feb;9(1):016006. doi: 10.1088/1741-2560/9/1/016006. Epub 2011 Dec 16. Dowden BR1, Franke

Properties of implanted electrodes for functional electrical stimulation. Ann Biomed Eng. 1991;19(3):303-16. Popovic D1, Gordon T, Rafuse VF, Prochazka A.

Recording sensory and motor information from peripheral nerves with Utah Slanted Electrode Arrays. Conf Proc IEEE Eng Med Biol Soc. 2011;2011:4641-4. doi: 10.1109/IEMBS.2011.6091149. Clark GA1, Ledbetter NM, Warren DJ, Harriso

Recruitment properties of intramuscular and nerve-trunk stimulating electrodes. IEEE Trans Rehabil Eng. 2000 Sep;8(3):276-85. Singh K1, Richmond FJ, Loeb GE.

Relationship between stimulus amplitude, stimulus frequency and neural damage during electrical stimulation of sciatic nerve of cat. Med Biol Eng Comput. 1995 May;33(3 Spec No):426-9. McCreery DB1, Agnew WF, Yuen TG, Bullara LA.

Restoration of use of paralyzed limb muscles using sensory nerve signals for state control of FES-assisted walking. IEEE Trans Rehabil Eng. 1999 Sep;7(3):289-300. Strange KD1, Hoffer JA.

Selective stimulation of cat sciatic nerve using an array of varying-length microelectrodes. J Neurophysiol. 2001 Apr;85(4):1585-94. Branner A1, Stein RB, Normann RA.

Selective stimulation of facial muscles with a penetrating electrode array in the feline model. Laryngoscope. 2017 Feb;127(2):460-465. doi: 10.1002/lary.26078. Epub 2016 Jun 16. Sahyouni R1, Bhatt J2, Djalilian HR2, Tang WC3, Middlebro

Selective stimulation of peripheral nerve fibers using dual intrafascicular electrodes. IEEE Trans Biomed Eng. 1993 May;40(5):492-4. Yoshida K1, Horch K.

Single- and multi-unit activity recorded from the surface of the dorsal root ganglia with non-penetrating electrode arrays. Conf Proc IEEE Eng Med Biol Soc. 2011;2011:6713-6. doi: 10.1109/IEMBS.2011.6091655. Gaunt RA1, Bruns TM, Cra

Single unit conduction velocities from averaged nerve cuff electrode records in freely moving cats. J Neurosci Methods. 1981 Oct;4(3):211-25. Hoffer JA, Loeb GE, Pratt CA.

Stability of the input-output properties of chronically implanted multiple contact nerve cuff stimulating electrodes. IEEE Trans Rehabil Eng. 1998 Dec;6(4):364-73. Grill WM1, Mortimer JT.

Stable long-term recordings from cat peripheral nerves. Brain Res. 1977 Jun 3;128(1):21-38. Stein RB, Nichols TR, Jhamandas J, Davis L, Charles D.

The design of and chronic tissue response to a composite nerve electrode with patterned stiffness. J Neural Eng. 2017 Jun;14(3):036022. doi: 10.1088/1741-2552/aa6632. Epub 2017 Mar 13. Freeberg MJ1, Stone MA, Triolo RJ, Tyler DJ.

The foreign body response to the Utah Slant Electrode Array in the cat sciatic nerve. Acta Biomater. 2014 Nov;10(11):4650-4660. doi: 10.1016/j.actbio.2014.07.010. Epub 2014 Jul 17. Christensen MB1, Pearce SM1, Ledbetter NM1, Warre

A Stretchable Microneedle Electrode Array for Stimulating and Measuring Intramuscular Electromyographic Activity. IEEE Trans Neural Syst Rehabil Eng. 2017 Sep;25(9):1440-1452. doi: 10.1109/TNSRE.2016.2629461. Epub 2016 Nov 16. G

Impedance properties of metal electrodes for chronic recording from mammalian nerves. IEEE Trans Biomed Eng. 1978 Nov;25(6):532-7. Stein RB, Charles D, Gordon T, Hoffer JA, Jhamandas J.

Chronic polyelectromyography in awake, unrestrained animals. Physiol Bohemoslov. 1978;27(5):485-92. Hník P, Kasicki S, Afelt Z, Vejsada R, Krekule I.

An implantable electrode design for both chronic in vivo nerve recording and axon stimulation in freely behaving crayfish. J Neurosci Methods. 2002 Jul 30;118(1):33-40. Gruhn M1, Rathmayer W.

Design and testing of an advanced implantable neuroprosthesis with myoelectric control. IEEE Trans Neural Syst Rehabil Eng. 2011 Feb;19(1):45-53. doi: 10.1109/TNSRE.2010.2079952. Epub 2010 Sep 27. Hart RL1, Bhadra N, Montague FW

Implantable electrode lead in a growing limb. IEEE Trans Rehabil Eng. 1999 Mar;7(1):35-45. Akers JM1, Smith BT, Betz RR.

Implantable multichannel wireless electromyography for prosthesis control. Conf Proc IEEE Eng Med Biol Soc. 2012;2012:1350-3. doi: 10.1109/EMBC.2012.6346188. McDonnall D1, Hiatt S, Smith C, Guillory KS.

Multielectrode spiral cuff for selective stimulation of nerve fibres. J Med Eng Technol. 1992 Sep-Oct;16(5):194-203. Rozman J1, Trlep M.

Regeneration of the radial nerve in a dog influenced by electrical stimulation. Pflugers Arch. 2000;439(3 Suppl):R184-6. Rozman J1, Zorko B, Seliskar A.

Selective recording of neuroelectric activity from the peripheral nerve. Pflugers Arch. 2000;440(5 Suppl):R157-9. Rozman J1, Zorko B, Seliskar A, Bunc M.

Selective recording of electroneurograms from the left vagus nerve of a dog during stimulation of cardiovascular or respiratory systems. Chin J Physiol. 2007 Oct 31;50(5):240-50. Rozman J1, Ribaric S.

Selective recording of electroneurograms from the sciatic nerve of a dog with multi-electrode spiral cuffs. Jpn J Physiol. 2000 Oct;50(5):509-14. Rozman J1, Zorko B, Bunc M.

Selective stimulation of the canine hypoglossal nerve using a multi-contact cuff electrode. Ann Biomed Eng. 2004 Apr;32(4):511-9. Yoo PB1, Sahin M, Durand DM.

Multielectrode spiral cuff for ordered and reversed activation of nerve fibres. J Biomed Eng. 1993 Mar;15(2):113-20. Rozman J1, Sovinec B, Trlep M, Zorko B.

The effects of electrical stimulation on denervated muscle using implantable electrodes. J Reconstr Microsurg. 1988 Jul;4(4):251-5, 257. Nemoto K1, Williams HB, Nemoto K, Lough J, Chiu RC.

Neural engineering--a new discipline for analyzing and interacting with the nervous system. Methods Inf Med. 2007;46(2):142-6. Durand DM1.

Regeneration electrode units: implants for recording from single peripheral nerve fibers in freely moving animals. Science. 1974 Feb 8;183(4124):547-9. Mannard A, Stein RB, Charles D.

An implantable myography electrode for recording muscle activity in freely moving small animals--a new technological approach. IEEE Trans Biomed Eng. 1982 Nov;29(11):730-6. Sigrist GV, Kleinebeckel DK.

Chronic cuff electrode recordings from walking Göttingen mini-pigs. Conf Proc IEEE Eng Med Biol Soc. 2011;2011:2280-3. doi: 10.1109/IEMBS.2011.6090574. Andersen MP1, Munch M, Jensen W, Sørensen P, Eder CF.

Biosafety assessment of an intra-neural electrode (TIME) following sub-chronic implantation in the median nerve of Göttingen minipigs. Int J Artif Organs. 2014 Jun;37(6):466-76. doi: 10.5301/ijao.5000342. Epub 2014 Jun 28. Kundu A1, W

New synthetic prosthesis for peripheral nerve injuries: an experimental pilot study. Surg Innov. 2013 Apr;20(2):171-5. doi: 10.1177/1553350612458546. Epub 2012 Sep 20. Uranüs S1, Bretthauer G, Nagele-Moser D, Saliba S, Tomasch G, F

Development, manufacturing and application of double-sided flexible implantable microelectrodes. Biomed Microdevices. 2014 Dec;16(6):837-50. doi: 10.1007/s10544-014-9887-8. Poppendieck W1, Sossalla A, Krob MO, Welsch C, Nguye

Easily-implantable electrodes for chronic recording of electromyogram activity in small fetuses. J Neurosci Methods. 1990 Jul;33(1):51-4. Cooke IR1, Brodecky V, Berger PJ.

Decoding individuated finger flexions with Implantable MyoElectric Sensors. Conf Proc IEEE Eng Med Biol Soc. 2008;2008:193-6. doi: 10.1109/IEMBS.2008.4649123. Baker JJ1, Yatsenko D, Schorsch JF, DeMichele GA, Troyk PR, Hutchinson

Chronic recording of EMG activity from large numbers of forelimb muscles in awake macaque monkeys. J Neurosci Methods. 2000 Mar 15;96(2):153-60. Park MC1, Belhaj-Saïf A, Cheney PD.

A new electrode configuration for recording electromyographic activity in behaving mice. J Neurosci Methods. 2005 Oct 15;148(1):36-42. Pearson KG1, Acharya H, Fouad K.

Design and evaluation of a chronic EMG multichannel detection system for long-term recordings of hindlimb muscles in behaving mice. J Electromyogr Kinesiol. 2013 Jun;23(3):531-9. doi: 10.1016/j.jelekin.2012.11.014. Epub 2013 Jan 29.

Long-term peripheral nerve and muscle recordings from normal and dystrophic mice. J Neurosci Methods. 1987 Jan;19(1):37-45. Milner TE, Hoffer JA.

OptoZIF Drive: a 3D printed implant and assembly tool package for neural recording and optical stimulation in freely moving mice. J Neural Eng. 2016 Dec;13(6):066013. Epub 2016 Oct 20. Freedman DS1, Schroeder JB, Telian GI, Zhang Z,

Fibre-selective recording from peripheral nerves using a multiple-contact cuff: report on pilot pig experiments. Conf Proc IEEE Eng Med Biol Soc. 2011;2011:3103-6. doi: 10.1109/IEMBS.2011.6090847. Schuettler M1, Seetohul V, Rijkhoff N

High-performance wireless powering for peripheral nerve neuromodulation systems. PLoS One. 2017 Oct 24;12(10):e0186698. doi: 10.1371/journal.pone.0186698. eCollection 2017. Tanabe Y1, Ho JS2, Liu J3, Liao SY4, Zhen Z4, Hsu S1, Sh

Selective stimulation of pig radial nerve: comparison of 12-polar and 18-polar cuff electrodes. Biomed Tech (Berl). 2002;47 Suppl 1 Pt 2:696-9. Schuettler M1, Riso RR, Dalmose A, Stefania D, Stieglitz T.

Selectivity for specific cardiovascular effects of vagal nerve stimulation with a multi-contact electrode cuff. IEEE Trans Neural Syst Rehabil Eng. 2013 Jan;21(1):32-6. doi: 10.1109/TNSRE.2012.2214058. Epub 2012 Sep 11. Ordelman SC1, Ko

Subchronic stimulation performance of transverse intrafascicular multichannel electrodes in the median nerve of the Göttingen minipig. Artif Organs. 2015 Feb;39(2):E36-48. doi: 10.1111/aor.12347. Epub 2014 Jul 23. Harreby KR1, Kundu

Stimulation selectivity of the “thin-film longitudinal intrafascicular electrode” (tfLIFE) and the “transverse intrafascicular multi-channel electrode” (TIME) in the large nerve animal model. IEEE Trans Neural Syst Rehabil Eng. 2014 Mar;22(2

A novel electrode design for chronic recording of electromyographic activity. J Neurosci Methods. 2006 Sep 30;156(1-2):228-30. Epub 2006 Apr 18.Shafford HL1, Strittmatter RR, Schadt JC.

A telemetry system to chronically record muscle activity in middle-sized animals. J Neurosci Methods. 2002 Mar 15;114(2):197-203. Langenbach GE1, van Ruijven LJ, van Eijden TM.

An implantable bi-directional wireless transmission system for transcutaneous biological signal recording. Physiol Meas. 2005 Feb;26(1):83-97. Liang CK1, Chen JJ, Chung CL, Cheng CL, Wang CC.

An implantable electrical interface for in vivo studies of the neuromuscular system. J Neurosci Methods. 1996 Dec;70(1):27-32. Koh TJ1, Leonard TR.

An implantable wireless system for muscle afferent recording from the sciatic nerve during functional electrical stimulation. Conf Proc IEEE Eng Med Biol Soc. 2013;2013:3610-3. doi: 10.1109/EMBC.2013.6610324. Song KI, Shon A, Chu JU,

Artefact reduction with alternative cuff configurations. IEEE Trans Biomed Eng. 2003 Oct;50(10):1160-6. Andreasen LN1, Struijk JJ.

Biocompatibility of a silicon based peripheral nerve electrode. Biomater Med Devices Artif Organs. 1982;10(2):103-22. Edell DJ, Churchill JN, Gourley IM.

Degeneration and regeneration in rabbit peripheral nerve with long-term nerve cuff electrode implant: a stereological study of myelinated and unmyelinated axons. Acta Neuropathol. 1998 Oct;96(4):365-78. Larsen JO1, Thomsen M, Hau

Estimation of peroneal and tibial afferent activity from a multichannel cuff placed on the sciatic nerve. Muscle Nerve. 2005 Nov;32(5):589-99. Cheng HS1, Ju MS, Lin CC.

Experiments on the development and use of a new generation of intra-neural electrodes to control robotic devices. Conf Proc IEEE Eng Med Biol Soc. 2006;1:2940-3. Micera S1, Sergi PN, Carpaneto J, Citi L, Bossi S, Koch KP, Hoffmann KP,

Fascicle-selectivity of an intraneural stimulation electrode in the rabbit sciatic nerve. IEEE Trans Biomed Eng. 2012 Jan;59(1):192-7. doi: 10.1109/TBME.2011.2169671. Epub 2011 Sep 26. Nielsen TN1, Sevcencu C, Struijk JJ.

Fuzzy control with amplitude/pulse-width modulation of nerve electrical stimulation for muscle force control. J Neural Eng. 2012 Apr;9(2):026026. doi: 10.1088/1741-2560/9/2/026026. Epub 2012 Mar 16. Lin CC1, Liu WC, Chan CC, Ju MS

High frequency block of selected axons using an implantable microstimulator. J Neurosci Methods. 2004 Mar 15;134(1):81-90. Peng CW1, Chen JJ, Lin CC, Poon PW, Liang CK, Lin KP.

Improved long-term recording of nerve signal by modified intrafascicular electrodes in rabbits. Microsurgery. 2008;28(3):173-8. doi: 10.1002/micr.20475. Jia X1, Zhen G, Puttgen A, Zhang J, Chen T.

Longitudinally implanted intrafascicular electrodes for stimulating and recording fascicular physioelectrical signals in the sciatic nerve of rabbits. Microsurgery. 2003;23(3):268-73. Zheng X1, Zhang J, Chen T, Chen Z.

Motion control of the rabbit ankle joint using a flat interface nerve electrode. Conf Proc IEEE Eng Med Biol Soc. 2009;2009:6789-92. doi: 10.1109/IEMBS.2009.5333979. Park H1, Durand DM.

Neural interfaces for regenerated nerve stimulation and recording. IEEE Trans Rehabil Eng. 1998 Dec;6(4):353-63. Dario P1, Garzella P, Toro M, Micera S, Alavi M, Meyer U, Valderrama E, Sebastiani L, Ghelarducci B, Mazzoni C, Pastacaldi

Neuromuscular stimulation selectivity of multiple-contact nerve cuff electrode arrays. Med Biol Eng Comput. 1995 May;33(3 Spec No):418-25. Sweeney JD1, Crawford NR, Brandon TA.

On cuff imbalance and tripolar ENG amplifier configurations. IEEE Trans Biomed Eng. 2005 Feb;52(2):314-20. Triantis IF1, Demosthenous A, Donaldson N.

On the use of wavelet denoising and spike sorting techniques to process electroneurographic signals recorded using intraneural electrodes. J Neurosci Methods. 2008 Jul 30;172(2):294-302. doi: 10.1016/j.jneumeth.2008.04.025. Epub 200

Peripheral nerve signal recording and processing for artificial limb control. Conf Proc IEEE Eng Med Biol Soc. 2010;2010:6206-9. doi: 10.1109/IEMBS.2010.5627735. Wodlinger B1, Durand DM.

Position-selective activation of peripheral nerve fibers with a cuff electrode. IEEE Trans Biomed Eng. 1996 Aug;43(8):851-6. Goodall EV1, de Breij JF, Holsheimer J.

Recording and stimulating properties of chronically implanted longitudinal intrafascicular electrodes in peripheral fascicles in an animal model. Microsurgery. 2008;28(3):203-9. doi: 10.1002/micr.20465. Zheng X1, Zhang J, Chen T, Chen Z.

Recovery of neural activity from nerve cuff electrodes. Conf Proc IEEE Eng Med Biol Soc. 2011;2011:4653-6. doi: 10.1109/IEMBS.2011.6091152. Wodlinger B1, Durand DM.

Selective recovery of fascicular activity in peripheral nerves. J Neural Eng. 2011 Oct;8(5):056005. doi: 10.1088/1741-2560/8/5/056005. Epub 2011 Aug 9. Wodlinger B1, Durand DM.

Acute peripheral nerve recording characteristics of polymer-based longitudinal intrafascicular electrodes. IEEE Trans Neural Syst Rehabil Eng. 2004 Sep;12(3):345-8. Lawrence SM1, Dhillon GS, Jensen W, Yoshida K, Horch KW.

The raccoon as an animal model for upper limb neural prosthetics. J Spinal Cord Med. 1996 Oct;19(4):234-41. Walter JS1, Griffith P, Scarpine V, Bidnar M, Dauzvardis M, Turner M, McLane J, Sweeney J, Robinson CJ.

A bipolar electrode for peripheral nerve stimulation. Brain Res Bull. 1979 May-Jun;4(3):421-2. Barone FC, Wayner MJ, Aguilar-Baturoni HU, Guevara-Aguilar R.

A fully implantable rodent neural stimulator. J Neural Eng. 2012 Feb;9(1):014001. doi: 10.1088/1741-2560/9/1/014001. Epub 2012 Jan 17. Perry DW1, Grayden DB, Shepherd RK, Fallon JB.

A method of nerve electrical stimulation by magnetic induction. Conf Proc IEEE Eng Med Biol Soc. 2009;2009:622-5. doi: 10.1109/IEMBS.2009.5333491. Zhang G1, Li Y, Huo X, Song T.

A micromachined silicon sieve electrode for nerve regeneration applications. IEEE Trans Biomed Eng. 1994 Apr;41(4):305-13. Akin T1, Najafi K, Smoke RH, Bradley RM.

A miniaturized cuff electrode for electrical stimulation of peripheral nerves in the freely moving rat. Brain Res Bull. 1995;37(5):551-4. Jellema T1, Teepen JL.

A New System and Paradigm for Chronic Stimulation of Denervated Rat Muscle. J Med Biol Eng. 2011;31(2):87-92. Willand MP1, Lopez JP, de Bruin H, Fahnestock M, Holmes M, Bain JR.

A regenerative microchannel device for recording multiple single-unit action potentials in awake, ambulatory animals Eur J Neurosci. 2016 Feb;43(3):474-85. doi: 10.1111/ejn.13080. Epub 2015 Oct 28.Srinivasan A1, Tipton J1, Tahilraman

A regenerative microchannel neural interface for recording from and stimulating peripheral axons in vivo. J Neural Eng. 2012 Feb;9(1):016010. doi: 10.1088/1741-2560/9/1/016010. Epub 2012 Jan 19. FitzGerald JJ1, Lago N, Benmerah S, S

A simple electrode for intact nerve stimulation and/or recording in semi-chronic rats. Pflugers Arch. 1983 Apr;397(1):68-9. Sauter JF, Berthoud HR, Jeanrenaud B.

A three-dimensional self-opening intraneural peripheral interface (SELINE). J Neural Eng. 2015 Feb;12(1):016016. doi: 10.1088/1741-2560/12/1/016016. Epub 2015 Jan 21. Cutrone A1, Del Valle J, Santos D, Badia J, Filippeschi C, Micera S,

A transverse intrafascicular multichannel electrode (TIME) to interface with the peripheral nerve. Biosens Bioelectron. 2010 Sep 15;26(1):62-9. doi: 10.1016/j.bios.2010.05.010. Epub 2010 May 11. Boretius T1, Badia J, Pascual-Font A, Schu

A wireless system with stimulation and recording capabilities for interfacing peripheral nerves in rodents. Conf Proc IEEE Eng Med Biol Soc. 2016 Aug;2016:4439-4442. doi: 10.1109/EMBC.2016.7591712. Schonle P, Michoud F, Brun N, Gu

Adjacent regenerative peripheral nerve interfaces produce phase-antagonist signals during voluntary walking in rats. J Neuroeng Rehabil. 2017 Apr 24;14(1):33. doi: 10.1186/s12984-017-0243-0. Ursu D1, Nedic A2, Urbanchek M2, Cedern

An integrated interface for peripheral neural system recording and stimulation: system design, electrical tests and in-vivo results. Carboni C1, Bisoni L2, Carta N2, Puddu R2, Raspopovic S3, Navarro X4, Raffo L2, Barbaro M2.

Assessment of biocompatibility of chronically implanted polyimide and platinum intrafascicular electrodes. IEEE Trans Biomed Eng. 2007 Feb;54(2):281-90. Lago N1, Yoshida K, Koch KP, Navarro X.

Awake evoked electromyography recording from the chronically implanted rat. Laryngoscope. 1994 Apr;104(4):420-5. Widick MH1, Tanabe T, Fortune S, Zealear DL.

Behavioral and cellular consequences of high-electrode count Utah Arrays chronically implanted in rat sciatic nerve. J Neural Eng. 2014 Aug;11(4):046027. doi: 10.1088/1741-2560/11/4/046027. Epub 2014 Jul 17. Wark HA1, Mathews KS,

Biocompatibility of chronically implanted transverse intrafascicular multichannel electrode (TIME) in the rat sciatic nerve. IEEE Trans Biomed Eng. 2011 Aug;58(8). doi: 10.1109/TBME.2011.2153850. Epub 2011 May 12. Badia J, Boretius T,

Biomechanical and functional variation in rat sciatic nerve following cuff electrode implantation. J Neuroeng Rehabil. 2014 Apr 23;11:73. doi: 10.1186/1743-0003-11-73. Restaino SM, Abliz E, Wachrathit K, Krauthamer V, Shah SB1.

Characterizing the reduction of stimulation artifact noise in a tripolar nerve cuff electrode by application of a conductive shield layer. Med Eng Phys. 2017 Feb;40:39-46. doi: 10.1016/j.medengphy.2016.11.010. Epub 2016 Dec 10. Sabetia

Chronic in-vivo testing of a 16-channel implantable wireless neural stimulator. Conf Proc IEEE Eng Med Biol Soc. 2015 Aug;2015:1017-20. doi: 10.1109/EMBC.2015.7318537. Bredeson S, Kanneganti A, Deku F, Cogan S, Romero-Ortega M,

Chronic multichannel neural recordings from soft regenerative microchannel electrodes during gait. Sci Rep. 2015 Sep 24;5:14363. doi: 10.1038/srep14363. Musick KM1, Rigosa J2,3, Narasimhan S1, Wurth S2, Capogrosso M2,3, Chew DJ

Chronic response of the rat sciatic nerve to the flat interface nerve electrode. Ann Biomed Eng. 2003 Jun;31(6):633-42. Tyler DJ1, Durand DM.

Chronic sensory-motor activity in behaving animals using regenerative multi-electrode interfaces. Conf Proc IEEE Eng Med Biol Soc. 2014;2014:1973-6. doi: 10.1109/EMBC.2014.6944000. Desai VH, Anand S, Tran M, Kanneganti A, Vasude

Chronically implanted epineural electrodes for repeated assessment of nerve conduction velocity and compound action potential amplitude in rodents. J Neurosci Methods. 2004 Jan 15;132(1):25-33. Murphy B1, Krieger C, Hoffer JA.

Comparative analysis of transverse intrafascicular multichannel, longitudinal intrafascicular and multipolar cuff electrodes for the selective stimulation of nerve fascicles. J Neural Eng. 2011 Jun;8(3):036023. doi: 10.1088/1741-2560/8/3/0

Comparison of intensity-dependent inhibition of spinal wide-dynamic range neurons by dorsal column and peripheral nerve stimulation in a rat model of neuropathic pain. Eur J Pain. 2014 Aug;18(7):978-88. doi: 10.1002/j.1532-2149.201

Conduction block of peripheral nerve using high-frequency alternating currents delivered through an intrafascicular electrode. Muscle Nerve. 2010 Jan;41(1):117-9. doi: 10.1002/mus.21496. Ackermann DM Jr1, Foldes EL, Bhadra N, Kilgo

Continuous Direct Current Nerve Block Using Multi Contact High Capacitance Electrodes. IEEE Trans Neural Syst Rehabil Eng. 2017 Jun;25(6):517-529. doi: 10.1109/TNSRE.2016.2589541. Epub 2016 Jul 9. Vrabec T, Bhadra N, Van Acker G,

Cuff electrode implantation around the sciatic nerve is associated with an upregulation of TNF-alpha and TGF-beta 1. J Neuroimmunol. 2005 Feb;159(1-2):75-86. Epub 2004 Nov 24. Vince V1, Thil MA, Gérard AC, Veraart C, Delbeke J, Coli

Delaying discharge after the stimulus significantly decreases muscle activation thresholds with small impact on the selectivity: an in vivo study using TIME. Med Biol Eng Comput. 2015 Apr;53(4):371-9. doi: 10.1007/s11517-015-1244-4. Ep

Design, in vitro and in vivo assessment of a multi-channel sieve electrode with integrated multiplexer. J Neural Eng. 2006 Jun;3(2):114-24. Epub 2006 Apr 18. Ramachandran A1, Schuettler M, Lago N, Doerge T, Koch KP, Navarro X, Hoffma

Development of a simple low noise amplifier for recording of sensory mass signals from peripheral nerves. Biomed Tech (Berl). 2009 Feb;54(1):1-7. doi: 10.1515/BMT.2009.001. Stieglitz T1, Klausmann D, Krueger TB.

Effects of chronic nerve cuff and intramuscular electrodes on rat triceps surae motor units. Neurosci Lett. 2001 Oct 12;312(1):1-4. Carp JS1, Chen XY, Sheikh H, Wolpaw JR.

Electrically stimulated signals from a long-term Regenerative Peripheral Nerve Interface. Conf Proc IEEE Eng Med Biol Soc. 2014;2014:1989-92. doi: 10.1109/EMBC.2014.6944004. Langhals NB, Woo SL, Moon JD, Larson JV, Leach MK, Cede

Endoneural selective stimulating using wire-microelectrode arrays. IEEE Trans Rehabil Eng. 1999 Dec;7(4):399-412. Smit JP1, Rutten WL, Boom HB.

Experimental validation of a hybrid computational model for selective stimulation using transverse intrafascicular multichannel electrodes. IEEE Trans Neural Syst Rehabil Eng. 2012 May;20(3):395-404. doi: 10.1109/TNSRE.2012.2189021.

Feasibility of Long-term Tibial Nerve Stimulation Using a Multi-contact and Wirelessly Powered Neurostimulation System Implanted in Rats. Urology. 2017 Apr;102:61-67. doi: 10.1016/j.urology.2016.11.013. Epub 2016 Nov 16. Moazzam

Flexible and self-adaptive neural ribbon with three-dimensional electrodes for sciatic nerve recording. Conf Proc IEEE Eng Med Biol Soc. 2015;2015:3157-60. doi: 10.1109/EMBC.2015.7319062. Xiang Z, Yen SC, Sheshadri S, Xue N, Lee SH,

Functional recordings from awake, behaving rodents through a microchannel based regenerative neural interface. J Neural Eng. 2015 Feb;12(1):016017. doi: 10.1088/1741-2560/12/1/016017. Epub 2015 Jan 21. Gore RK1, Choi Y, Bellamk

High density penetrating electrode arrays for autonomic nerves. Conf Proc IEEE Eng Med Biol Soc. 2016 Aug;2016:2802-2805. doi: 10.1109/EMBC.2016.7591312. Burns J, Yee-Hsee Hsieh, Mueller A, Chevallier J, Sriram TS, Lewis SJ, Chew D

Immunohistochemical characterization of axonal sprouting and reactive tissue changes after long-term implantation of a polyimide sieve electrode to the transected adult rat sciatic nerve. Biomaterials. 2001 Sep;22(17):2333-43. Klinge P

Implantable microelectrodes with new electro-conductive materials for recording sympathetic neural discharge. Jpn J Physiol. 2003 Feb;53(1):61-4. Matsukawa K1, Komine H, Tsuchimochi H, Murata J, Yonezawa Y, Kondo K, Seki Y.

Improvement of signal-to-interference ratio and signal-to-noise ratio in nerve cuff electrode systems. Physiol Meas. 2012 Jun;33(6):943-67. doi: 10.1088/0967-3334/33/6/943. Epub 2012 May 3. Chu JU1, Song KI, Han S, Lee SH, Kim J, Kan

In vivo characterization of regenerative peripheral nerve interface function. J Neural Eng. 2016 Apr;13(2):026012. doi: 10.1088/1741-2560/13/2/026012. Epub 2016 Feb 9. Ursu DC1, Urbanchek MG, Nedic A, Cederna PS, Gillespie RB.

In vivo testing of a 3D bifurcating microchannel scaffold inducing separation of regenerating axon bundles in peripheral nerves. J Neural Eng. 2013 Dec;10(6):066018. doi: 10.1088/1741-2560/10/6/066018. Epub 2013 Nov 27. Stoyanova I

Long micro-channel electrode arrays: a novel type of regenerative peripheral nerve interface. IEEE Trans Neural Syst Rehabil Eng. 2009 Oct;17(5):454-60. doi: 10.1109/TNSRE.2009.2031241. Epub 2009 Sep 9. Lacour SP1, Fitzgerald JJ, Lago

Long term assessment of axonal regeneration through polyimide regenerative electrodes to interface the peripheral nerve. Biomaterials. 2005 May;26(14):2021-31. Lago N1, Ceballos D, Rodríguez FJ, Stieglitz T, Navarro X.

Long term chronic recordings from peripheral sensory fibers using a sieve electrode array. J Neurosci Methods. 1997 May 16;73(2):177-86. Bradley RM1, Cao X, Akin T, Najafi K.

Long-term usability and bio-integration of polyimide-based intra-neural stimulating electrodes. Biomaterials. 2017 Apr;122:114-129. doi: 10.1016/j.biomaterials.2017.01.014. Epub 2017 Jan 13. Wurth S1, Capogrosso M2, Raspopovic S2, C

Microchannel neural interface manufacture by stacking silicone and metal foil laminae. J Neural Eng. 2016 Jun;13(3):034001. doi: 10.1088/1741-2560/13/3/034001. Epub 2016 Mar 22. Lancashire HT1, Vanhoestenberghe A, Pendegrass C

Microtube-based electrode arrays for low invasive extracellular recording with a high signal-to-noise ratio. Biomed Microdevices. 2010 Feb;12(1):41-8. doi: 10.1007/s10544-009-9356-y. Takei K1, Kawano T, Kawashima T, Sawada K, Kanek

Multifunctional hydrogel coatings on the surface of neural cuff electrode for improving electrode-nerve tissue interfaces. Acta Biomater. 2016 Jul 15;39:25-33. doi: 10.1016/j.actbio.2016.05.009. Epub 2016 May 6. Heo DN1, Song SJ2, Kim

Neurobiological assessment of regenerative electrodes for bidirectional interfacing injured peripheral nerves. IEEE Trans Biomed Eng. 2007 Jun;54(6 Pt 1):1129-37. Lago N1, Udina E, Ramachandran A, Navarro X.

Neuromuscular electrical stimulation induced forelimb movement in a rodent model. J Neurosci Methods. 2008 Jan 30;167(2):317-26. Epub 2007 Aug 8. Kanchiku T1, Lynskey JV, Protas D, Abbas JJ, Jung R.

Polyimide cuff electrodes for peripheral nerve stimulation. J Neurosci Methods. 2000 Jun 1;98(2):105-18. Rodríguez FJ1, Ceballos D, Schüttler M, Valero A, Valderrama E, Stieglitz T, Navarro X.

Rapid prototyping of flexible intrafascicular electrode arrays by picosecond laser structuring. J Neural Eng. 2017 Dec;14(6):066016. doi: 10.1088/1741-2552/aa7eea. Mueller M1, de la Oliva N, Del Valle J, Delgado-Martínez I, Navarro X, Sti

Regeneration microelectrode array for peripheral nerve recording and stimulation. IEEE Trans Biomed Eng. 1992 Sep;39(9):893-902. Kovacs GT1, Storment CW, Rosen JM.

Regenerative Peripheral Nerve Interface for Prostheses Control: Electrode Comparison. J Reconstr Microsurg. 2016 Mar;32(3):194-9. doi: 10.1055/s-0035-1565248. Epub 2015 Oct 26. Sando IC1, Leach MK1, Woo SL1, Moon JD1, Cederna

Regenerative peripheral nerve interface viability and signal transduction with an implanted electrode. Plast Reconstr Surg. 2014 Jun;133(6):1380-94. doi: 10.1097/PRS.0000000000000168. Kung TA1, Langhals NB, Martin DC, Johnson PJ, C

Regenerative scaffold electrodes for peripheral nerve interfacing. IEEE Trans Neural Syst Rehabil Eng. 2013 Jul;21(4):554-66. doi: 10.1109/TNSRE.2012.2217352. Epub 2012 Sep 28. Clements IP1, Mukhatyar VJ, Srinivasan A, Bentley JT, And

Robust and real-time monitoring of nerve regeneration using implantable flexible microelectrode array. Biosens Bioelectron. 2009 Mar 15;24(7):1883-7. doi: 10.1016/j.bios.2008.09.034. Epub 2008 Oct 17. Kim YH1, Lee C, Ahn KM, Lee M,

Rodent model for assessing the long term safety and performance of peripheral nerve recording electrodes. J Neural Eng. 2017 Feb;14(1):016008. doi: 10.1088/1741-2552/14/1/016008. Epub 2016 Dec 9. Vasudevan S1, Patel K, Welle C.

Selective fascicular stimulation of the rat sciatic nerve with multipolar polyimide cuff electrodes. Restor Neurol Neurosci. 2001;18(1):9-21. Navarro X1, Valderrama E, Stieglitz T, Schüttler M.

Self-organization of "fibro-axonal" composite tissue around unmodified metallic micro-electrodes can form a functioning interface with a peripheral nerve: A new direction for creating long-term neural interfaces. Muscle Nerve. 2016 Ma

Sensitivity and selectivity of intraneural stimulation using a silicon electrode array. IEEE Trans Biomed Eng. 1991 Feb;38(2):192-8. Rutten WL1, van Wier HJ, Put JH.

Serial assessment of functional recovery following nerve injury using implantable thin-film wireless nerve stimulators. Muscle Nerve. 2016 Dec;54(6):1114-1119. doi: 10.1002/mus.25153. Epub 2016 Oct 6. Gamble P1, Stephen M1, MacEw

Serial estimation of motor unit numbers using an implantable system following nerve injury and repair in rats. Conf Proc IEEE Eng Med Biol Soc. 2016 Aug;2016:323-326. doi: 10.1109/EMBC.2016.7590705. Willand MP, Catapano J.

Silicon-substrate microelectrode arrays for parallel recording of neural activity in peripheral and cranial nerves. IEEE Trans Biomed Eng. 1994 Jun;41(6):567-77. Kovacs GT1, Storment CW, Halks-Miller M, Belczynski CR Jr, Della Santina CC,

Simultaneous Recordings of Central and Peripheral Bioelectrical Signals in a Freely Moving Rodent. Biol Pharm Bull. 2017;40(5):711-715. doi: 10.1248/bpb.b17-00070. Sasaki T1, Nishimura Y1, Ikegaya Y1,2.

Spatial and Functional Selectivity of Peripheral Nerve Signal Recording With the Transversal Intrafascicular Multichannel Electrode (TIME). IEEE Trans Neural Syst Rehabil Eng. 2016 Jan;24(1):20-7. doi: 10.1109/TNSRE.2015.2440768. Epub

Stimulation and recording from regenerated peripheral nerves through polyimide sieve electrodes. J Peripher Nerv Syst. 1998;3(2):91-101. Navarro X1, Calvet S, Rodríguez FJ, Stieglitz T, Blau C, Butí M, Valderrama E, Meyer JU.

Suppression of scarring in peripheral nerve implants by drug elution. J Neural Eng. 2016 Apr;13(2):026006. doi: 10.1088/1741-2560/13/2/026006. Epub 2016 Jan 29. FitzGerald JJ1.

Time course of tissue remodelling and electrophysiology in the rat sciatic nerve after spiral cuff electrode implantation. J Neuroimmunol. 2007 Apr;185(1-2):103-14. Epub 2007 Mar 6. Thil MA1, Duy DT, Colin IM, Delbeke J.

Use of tripolar electrodes for minimization of current spread in uncut peripheral nerve stimulation. Neurosci Res. 2009 May;64(1):63-6. doi: 10.1016/j.neures.2009.01.016. Epub 2009 Feb 7. Ohsawa I1, Inui K.

A new high-density (25 electrodes/mm²) penetrating microelectrode array for recording and stimulating sub-millimeter neuroanatomical structures. J Neural Eng. 2013 Aug;10(4):045003. doi: 10.1088/1741-2560/10/4/045003. Epub 201

An intrafascicular electrode for recording of action potentials in peripheral nerves. Ann Biomed Eng. 1989;17(4):397-410. Malagodi MS1, Horch KW, Schoenberg AA.

Prosthesis Control with an Implantable Multichannel Wireless Electromyography System for High-Level Amputees: A Large-Animal Study. Plast Reconstr Surg. 2016 Jan;137(1):153-62. doi: 10.1097/PRS.0000000000001926. Bergmeister K

Morphologic and functional evaluation of peripheral nerve fibers regenerated through polyimide sieve electrodes over long-term implantation. J Biomed Mater Res. 2002 Jun 15;60(4):517-28. Ceballos D1, Valero-Cabré A, Valderrama E, S

Long-term decoding of movement force and direction with a wireless myoelectric implant. J Neural Eng. 2016 Feb;13(1):016002. doi: 10.1088/1741-2560/13/1/016002. Epub 2015 Dec 8. Morel P1, Ferrea E, Taghizadeh-Sarshouri B, Audí J

Methods for chronic recording of EMG activity from large numbers of hindlimb muscles in awake rhesus macaques. J Neurosci Methods. 2010 Jun 15;189(2):153-61. doi: 10.1016/j.jneumeth.2010.03.011. Epub 2010 Mar 25. Hudson HM1

Acquisition of myoelectric signals to control a hand prosthesis with implantable epimysial electrodes. Conf Proc IEEE Eng Med Biol Soc. 2010;2010:5070-3. doi: 10.1109/IEMBS.2010.5626226. Ruff R1, Poppendieck W, Gail A, Westendorff S

Chronic recording of hand prosthesis control signals via a regenerative peripheral nerve interface in a rhesus macaque. J Neural Eng. 2016 Aug;13(4):046007. doi: 10.1088/1741-2560/13/4/046007. Epub 2016 Jun 1. Irwin ZT1, Schroeder K

Continuous detection and decoding of dexterous finger flexions with implantable myoelectric sensors. IEEE Trans Neural Syst Rehabil Eng. 2010 Aug;18(4):424-32. doi: 10.1109/TNSRE.2010.2047590. Epub 2010 Apr 8. Baker JJ1, Scheme E,

Intrafascicular stimulation of monkey arm nerves evokes coordinated grasp and sensory responses. J Neurophysiol. 2013 Jan;109(2):580-90. doi: 10.1152/jn.00688.2011. Epub 2012 Oct 17. Ledbetter NM1, Ethier C, Oby ER, Hiatt SD, Wild

The use of a bone-anchored device as a hard-wired conduit for transmitting EMG signals from implanted muscle electrodes. IEEE Trans Biomed Eng. 2013 Jun;60(6):1654-9. doi: 10.1109/TBME.2013.2241060. Epub 2013 Jan 23. Al-Ajam Y1

A micro-scale printable nanoclip for electrical stimulation and recording in small nerves. J Neural Eng. 2017 Jun;14(3):036006. doi: 10.1088/1741-2552/aa5a5b. Epub 2017 Mar 21.Lissandrello CA1, Gillis WF, Shen J, Pearre BW, Vitale F, Pa

Characterization of signals and noise rejection with bipolar longitudinal intrafascicular electrodes. IEEE Trans Biomed Eng. 1999 Feb;46(2):226-34. Yoshida K1, Stein RB.
